# Supplementary material for: Causal events enter awareness faster than non-causal events
Source: PeerJ. 2017 Jan 26;5:e2932. doi: 10.7717/peerj.2932 (PMC5274517; doi:10.7717/peerj.2932)
Supplement: Supplemental Information 1 [file peerj-05-2932-s001.docx]

**Supplementary Materials**

Causal events enter awareness faster than non-causal events

Pieter Moors, Johan Wagemans, Lee de-Wit

In this Supplementary Material, we report on two analyses that assess the robustness of the effects we reported in the main text, by analyzing how sensitive they are to our exclusion criteria. In our main analyses of Experiments 1 and 2, we used the following exclusion criteria:

- Suppression times shorter than 800 ms were deleted because they reflect processing before any event occurred.
- Outliers were defined as suppression times deviating more than three standard deviations from the participants’ mean suppression time, and were deleted (outliers excluded/included).
- Participants who responded on more than 10% of the catch trials were deleted (catch excluded/included).
- If more than 10% of suppression times had to be deleted due to too fast responses, the participant was completely removed from the data set (too fast excluded/included).

The first criterion is one that is related to the question we asked. Therefore, in these supplementary analyses, we always removed the “too fast responses” from our data set. For all other criteria, we assessed whether applying them or not changed the outcome of the analysis. We focused specifically on the effect of event type, because this was of most interest to us. Therefore, in the following tables, we report whether the effect of event type remained after applying different combinations of exclusion criteria.

As can be derived from Table S1, very strong evidence was obtained for an effect of event type (launch vs. pass) across all different exclusion criteria. Table S2 depicts the same pattern of results, but now for the launch vs. pass comparison in Experiment 2. Again, strong evidence in favor of an effect of event type was observed across all different exclusion criteria. Table S3 shows that, across the exclusion criteria, strong evidence in favor of the absence of a difference between the pass and pseudolaunch events is obtained. Last, as can be derived from Table S4, moderate to strong evidence was obtained in favor of a difference between launch and pseudolaunch events across all different exclusion criteria.

In sum, this supplementary analysis shows whether or not (some of our) exclusion criteria are applied does not change the general pattern of our results. In fact, applying all exclusion criteria yielded the most *conservative* estimate of the strength of evidence for the effects of event type in both Experiments 1 and 2, rather than resulting in less strong evidence when those criteria were not applied.

*Table S1. Robustness analysis for Experiment 1.*

|  | **Outliers excluded** | | **Outliers included** | |
| --- | --- | --- | --- | --- |
|  | **Catch excluded** | **Catch included** | **Catch excluded** | **Catch included** |
| **Too fast excluded** | 32818 (2.04%) | 162343 (8.07%) | 218830 (5.51%) | 1556462 (1.24%) |
| **Too fast included** | 26875 (2.08%) | 117253 (0.75%) | 490794 (1.55% | 3660335 (1.79%) |

*Note.* All reported values are Bayes factors for a model including an effect of event type in the numerator and an empty model in the denominator. Percentages in brackets indicate the error associated with estimating the Bayes factor.

*Table S2. Robustness analysis for Experiment 2: launch vs. pass*

|  | **Outliers excluded** | | **Outliers included** | |
| --- | --- | --- | --- | --- |
|  | **Catch excluded** | **Catch included** | **Catch excluded** | **Catch included** |
| **Too fast excluded** | 256 (1.57%) | 2078 (2.89%) | 2605 (0.95%) | 15703 (1.13%) |
| **Too fast included** | 404 (2.14%) | 3811 (4.35%) | 4234 (3.12%) | 40039 (1.52%) |

*Note.* All reported values are Bayes factors for a model including an effect of event type (launch vs. pass) in the numerator and an empty model in the denominator. Percentages in brackets indicate the error associated with estimating the Bayes factor.

*Table S3. Robustness analysis for Experiment 2: pass vs. pseudolaunch*

|  | **Outliers excluded** | | **Outliers included** | |
| --- | --- | --- | --- | --- |
|  | **Catch excluded** | **Catch included** | **Catch excluded** | **Catch included** |
| **Too fast excluded** | 0.07 (1.15%) | 0.04 (0.91%) | 0.06 (1.04%) | 0.04 (1.39%) |
| **Too fast included** | 0.05 (0.92%) | 0.04 (1.74%) | 0.05 (8.3%) | 0.04 (3.66%) |

*Note.* All reported values are Bayes factors for a model including an effect of event type (pass vs. pseudo-launch) in the numerator and an empty model in the denominator. Percentages in brackets indicate the error associated with estimating the Bayes factor.

*Table S4. Robustness analysis for Experiment 2: launch vs. pseudolaunch*

|  | **Outliers excluded** | | **Outliers included** | |
| --- | --- | --- | --- | --- |
|  | **Catch excluded** | **Catch included** | **Catch excluded** | **Catch included** |
| **Too fast excluded** | 3 (1.04%) | 723 (1.73%) | 41 (1.74%) | 13158 (1.19%) |
| **Too fast included** | 16 (1.6%) | 3833 (1.63%) | 283 (1.27%) | 103798 (1.17%) |

*Note.* All reported values are Bayes factors for a model including an effect of event type (launch vs. pseudolaunch) in the numerator and an empty model in the denominator. Percentages in brackets indicate the error associated with estimating the Bayes factor.
